# Effectiveness and Overall Safety of NutropinAq^®^ for Growth Hormone Deficiency and Other Paediatric Growth Hormone Disorders: Completion of the International Cooperative Growth Study, NutropinAq^®^ European Registry (iNCGS)

**DOI:** 10.3389/fendo.2021.676083

**Published:** 2021-05-25

**Authors:** Regis Coutant, Jordi Bosch Muñoz, Cristina Patricia Dumitrescu, Dirk Schnabel, Caroline Sert, Valerie Perrot, Mehul Dattani

**Affiliations:** ^1^ Pediatric Endocrinology, Centre Hospitalier Universitaire d’Angers, Angers, France; ^2^ Pediatric Endocrinology, Hospital Arnau de Vilanova, Lleida, Spain; ^3^ Pediatric Endocrinology, Constantin Ion Parhon (C.I.) Parhon National Institute of Endocrinology, Bucharest, Romania; ^4^ Center for Chronic Sick Children, Pediatric Endocrinology, Charitè, University Medicine, Berlin, Germany; ^5^ Ipsen, Boulogne-Billancourt, France; ^6^ University College London (UCL) Great Ormond Street (GOS) Institute of Child Health and Great Ormond Street Hospital for Children, London, United Kingdom

**Keywords:** NutropinAq^®^ (somatropin), rhGH, recombinant human GH, growth hormone deficiency, paediatric GH disorders, safety

## Abstract

**Objective:**

The International Cooperative Growth Study, NutropinAq^®^ European Registry (iNCGS) (NCT00455728) monitored long-term safety and effectiveness of recombinant human growth hormone (rhGH; NutropinAq^®^ [somatropin]) in paediatric growth disorders.

**Methods:**

Open-label, non-interventional, post-marketing surveillance study recruiting children with growth disorders. Endpoints included gain in height standard deviation score (SDS), adult height, and occurrence of adverse events (AEs).

**Results:**

2792 patients were enrolled. 2082 patients (74.6%) had growth hormone deficiency (GHD), which was isolated idiopathic in 1825 patients (87.7%). Non-GHD diagnoses included Turner syndrome (TS) (n=199), chronic renal insufficiency (CRI) (n=10), other non-GHD (n=498), and missing data for three participants. Improvements from baseline height SDS occurred at all time points to Month 132, and in all subgroups by disease aetiology. At Month 12, mean (95% CI) change in height SDS by aetiology was: idiopathic GHD 0.63 (0.61;0.66), organic GHD 0.71 (0.62;0.80), TS 0.59 (0.53; 0.65), CRI 0.54 (-0.49;1.56), and other non-GHD 0.64 (0.59;0.69). Mean height ( ± SD) at the last visit among the 235 patients with adult or near-adult height recorded was 154.0 cm ( ± 8.0) for girls and 166.7 cm ( ± 8.0) for boys. The most frequent biological and clinical non-serious drug-related AEs were increased insulin-like growth factor concentrations (314 events) and injection site haematoma (99 events). Serious AEs related to rhGH according to investigators were reported (n=30); the most frequent were scoliosis (4 events), epiphysiolysis (3 events), and strabismus (2 events).

**Conclusions:**

There was an improvement in mean height SDS in all aetiology subgroups after rhGH treatment. No new safety concerns were identified.

## Introduction

Growth hormone deficiency (GHD) is characterised by low growth velocity, sometimes after a period of normal growth, and short stature relative to the child’s chronological age, gender and pubertal stage (1). GHD may be congenital, presenting either in the neonatal period or later in childhood (2). or acquired in childhood due to brain trauma, brain tumour, infiltrations, or radiation treatment (3, 4). A substantial proportion of GH-deficient children may have idiopathic GHD, despite the large number of potential aetiologies (4, 5).

GHD is treated by GH therapy (6), with the aims of promoting linear growth, achieving expected adult height, restoring body composition and improving quality of life (7, 8). GH replacement therapy has previously demonstrated efficacy in children treated for GHD and other conditions associated with short stature such as Turner syndrome (TS), chronic renal insufficiency (CRI), and children born small for gestational age (9–12), with patients with GHD reaching near-adult heights of -0.4 standard deviation (SD) in one study (9). GH therapy has been shown to have a good safety profile (10, 12). A number of registries have assessed the safety and efficacy/effectiveness of the various GH therapies available, in various geographies and for the conditions outlined above (13–17).

Recombinant human growth hormone (rhGH; NutropinAq^®^, Ipsen Pharma, France; Genentech Inc, USA) is a solution for daily subcutaneous (SC) injection containing 10 mg (30 IU) of somatropin, a synthetic form of human GH produced by recombinant DNA technology (18). In the paediatric population, NutropinAq^®^ is licensed for the long-term treatment of growth failure due to inadequate endogenous GH secretion, the long-term treatment of girls aged ≥2 years with growth failure associated with TS, and the treatment of prepubertal children with growth failure associated with CRI up to the time of renal transplantation (18).

The rationale for the International Cooperative Growth Study, NutropinAq^®^ European Registry (iNCGS) was to collect long-term data on effectiveness and safety using NutropinAq^®^ under usual clinical conditions for the treatment of paediatric growth disorders for which GH is indicated. The objective of this manuscript is to present the final data, from the close of the registry in December 2016, and complementary additional analysis of breakdown by country and effectiveness parameters according to peak GH, as previously reported in preliminary form (19, 20).

## Materials and Methods

Approval was received from all relevant Ethics Committees and Institutional Review Boards before the study was initiated; written informed consent was obtained from the parents or legal guardians and the patient (where applicable) before enrolment and data collection.

### Trial Design

The iNCGS was an international, multicentre, open-label, non-interventional post-marketing surveillance study (ClinicalTrials.gov NCT00455728) that was initiated in 2005 and conducted at centres in seven European countries (France, Germany, Austria, Italy, Spain, UK and Romania). Data were collected until December 2016. The primary objective of the study was to collect long-term safety information on rhGH during treatment of paediatric growth disorders.

### Patients

All children presenting at the participating centres with growth disorders, for whom GH therapy was indicated and initiated (treatment-naïve group), and those who were already receiving therapy with rhGH, were eligible for enrolment into the study and were assessed throughout their course of treatment. The decision to prescribe rhGH was made independently of the decision to enrol the patient in the study.

Patients were excluded from the study if they were currently participating in any clinical trial for growth retardation, were currently using GH preparations other than NutropinAq^®^, were receiving insulin-like growth factor-1 (IGF-1), if treatment with rhGH was contraindicated, they had closed epiphyses, active neoplasia or active intracranial tumours, or they had received anti-tumour therapy within the past 12 months before instituting GH therapy. There were no predefined reasons for patient discontinuation as this was a non-interventional study.

### Dosing

The administered dose of rhGH was in accordance with the prescribing information and clinical practice. Dosing was individualised based on the Investigator’s clinical judgement.

According to the NutropinAq^®^ prescribing information, doses of 0.025–0.035 mg/kg bodyweight (in the treatment of growth failure due to GH deficiency) or up to 0.05 mg/kg bodyweight (in the treatment of growth failure associated with TS or CRI) are given daily to children and adolescents *via* SC injection until their epiphyses are closed or, in patients with CRI, until the time of renal transplantation.

### Assessments and Outcomes

Data could be collected until the patients reached adult height, i.e., epiphyseal closure. During the enrolment visit, the Investigator checked patient eligibility, obtained the signed Informed Consent Forms, and recorded demographic information and family medical history, disease history and aetiology. Treatment with rhGH was initiated at the baseline visit, with the exception of patients who had already begun treatment before enrolment. At the baseline visit, relevant medical information and history, treatment dose, schedule of rhGH and adverse events (AEs) were collected. Patient assessments included height, weight, body mass index (BMI), pubertal status, concomitant medications, vital signs, laboratory measurements and left hand/wrist X-ray for bone age determination. Information was added retrospectively for patients who had begun rhGH treatment before enrolment. Follow-up visits were conducted according to current practice at the participating centre, and evaluations at these visits included details of treatment administration, patient assessments (injection site administration, laboratory tests, vital signs, anthropometry, pubertal status, bone age, compliance) and collection of AEs.

The effectiveness and safety endpoints included height SD score (SDS), height velocity (cm/year), predicted adult height [PAH; calculated using the Bayley-Pinneau method (21)], near adult height (height velocity <1cm/year at last follow-up and after ≥1 year of treatment) and adult height (epiphyseal closure), and the description and incidence of any serious treatment-emergent AEs (TEAEs), the incidence of any TEAEs considered by the Investigator to be related to rhGH, and the incidence of any TEAEs leading to withdrawal. Investigators were asked to report non-serious related AEs and all serious AEs (regardless of relationship to study treatment). Other endpoints included change from baseline in body mass index SDS and IGF-1 concentrations, pubertal status and laboratory test results and/or vital signs data. The primary outcome measure was the number of patients with AEs.

The reference growth charts used were those used as standard in the participating countries: Sempe (22) and Programme National Nutrition Santé (PNNS) reference data in France, Spain, Italy and Romania; reference data for the UK (23); and KiGGS (24) and Kromeyer (25) reference data for Germany and Austria.

Treatment duration for each patient was according to the Investigator’s judgement based on the clinical needs of the individual patient and their responsiveness. Patients could be assessed throughout their entire course of treatment and data could be collected until the patient reached adult height.

### Statistics

The Enrolled population included all patients who were fully informed about the study, who gave written informed consent to participate and with at least one dataset collected in the registry. The Treated population included all enrolled patients who received at least one rhGH injection. The Registry population included all enrolled patients who completed at least one follow-up visit and who received at least one rhGH injection. The Completed population included all patients of the Registry population who attained adult height (with closed epiphyses/reached adult height as reason for end of registry) or had attained near-adult height (defined as height velocity <1 cm/year at last follow-up and after at least 12 months of treatment at registry closure). The Safety population included all patients who received at least one rhGH injection and with at least one follow-up visit or follow-up safety data.

Statistical analysis was performed using Statistical Analysis System (SAS^®^) Version 9.4 (SAS Institute Incorporated, Cary, NC, USA). Linear regression analyses were performed to identify predictive factors for change from baseline in height SDS and height velocity at Month 12, Month 24, Month 36 or Month 48 as well as final height SDS in treatment-naïve patients in the Registry population. For descriptive variables, the mean, SD, two-sided 95% CI of the mean, the median, quartiles Q1 and Q3, and the minimum and maximum were recorded. For qualitative variables, the frequency percentage of each modality and the two-sided 95% CI were calculated.

Covariates were aetiology, pubertal status, biological mother’s height (cm), biological father’s height (cm), birth length (cm), target height (cm), height (SDS) and weight (SDS) at baseline, starting dose (mg/kg/day), and age at first rhGH intake (years). Mean dose (mg/kg/day) and dose adaptation (defined as a change of at least 10% of the dose) during the first and subsequent years were additional covariates at Months 12-48. The threshold for the univariate analyses was p<0.2; the definition of significance for the multivariate analyses was p<0.05.

Complementary additional analyses were conducted as follows: height SDS at Month 12 for five peak plasma GH levels at baseline were compared by ANOVA, with a 5% level of significance. Data for breakdown by countries (up to the cut-off date of December 2014) were analysed descriptively on baseline characteristics, dose and duration of treatment.

## Results

### Patient Populations and Disposition

**Figure 1:**
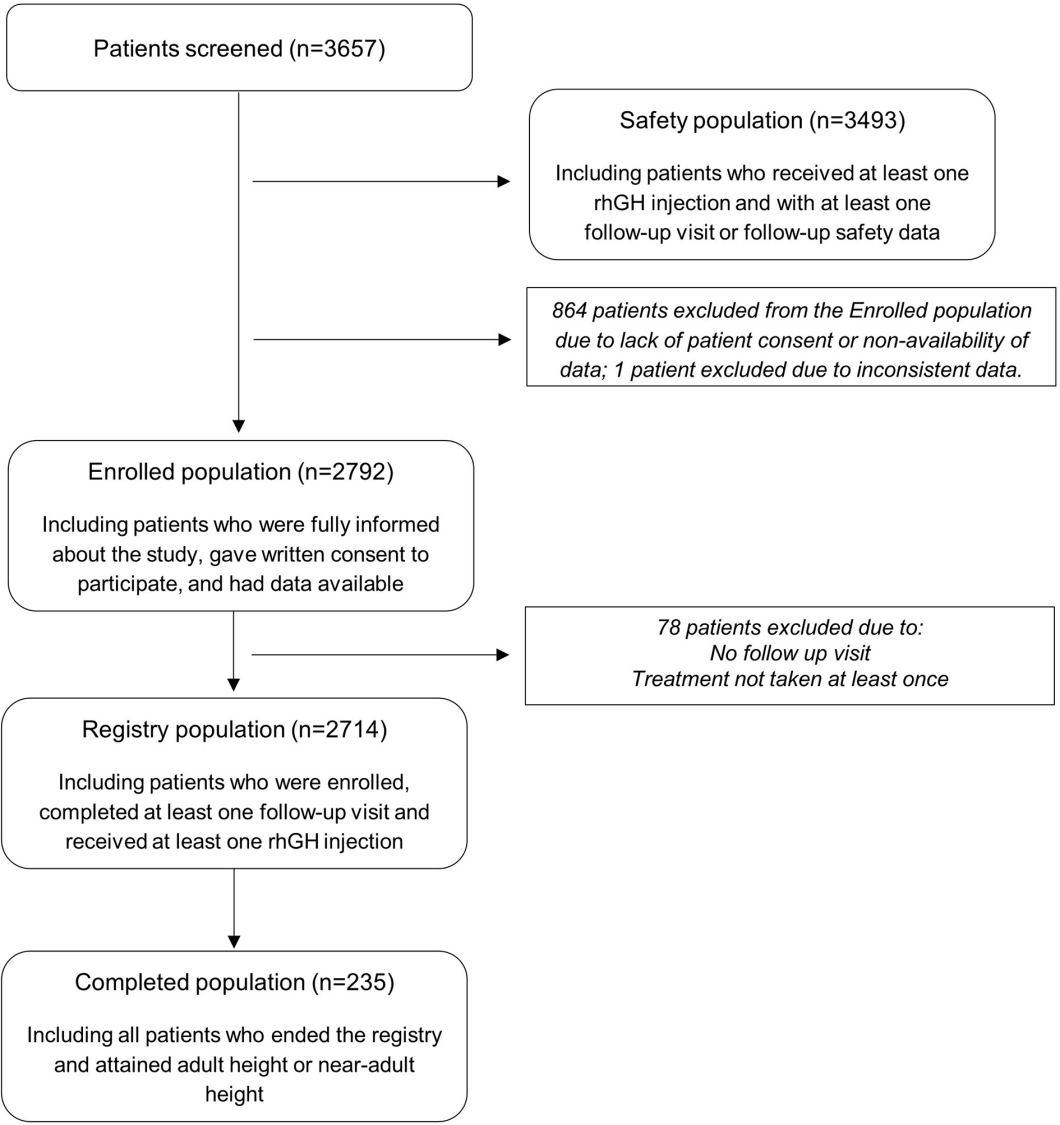
International Cooperative Growth Study, NutropinAq^®^ European Registry participant flow chart.

A total of 3657 patients were screened for the study at 166 centres; 864 were excluded from the Enrolled population due to lack of patient consent or non-availability of data, and one additional patient was excluded due to inconsistent data. The 2792 patients in the Enrolled population were included from 118 centres. The population distribution is summarised in **Figure 1**. Baseline characteristics are presented in **Table 1**, and the disposition of subgroups is summarised in **Table 2**. Most patients were treatment-naïve, the mean (±SD) age at diagnosis was 7.7 (±4.1) years and the mean ±SD) age at first rhGH intake was 9.5 (±3.6) years, and therefore, the mean delay between diagnosis and treatment initiation was >1 year. Nearly 50% of patients started treatment after 10 years of age. Approximately 60% of patients were boys (**Table 1**). Most patients had GHD, of which most had idiopathic GHD (**Table 2**).

**Table 1:** Baseline characteristics – Enrolled population treatment naïve.

Characteristic	All GHD (N=1801)	Organic GHD (N=214)	Idiopathic GHD (N=1585)	TS (N=165)^a^	CRI (N=9)	Other non-GHD (N=406)
**Gender**						
n	1801	214	1585	165	9	406
Boys	1192 (66.2)	133 (62.1)	1057 (66.7)	0	6 (66.7)	237 (58.4)
Girls	609 (33.8)	81 (37.9)	528 (33.3)	165 (100)	3 (33.3)	169 (41.6)
**Age at diagnosis (years)**						
n	1780	209	1569	165	9	402
Mean (SD)	8.4 (3.6)	8.0 (3.9)	8.5 (3.6)	5.2 (4.7)	5.0 (4.7)	6.2 (4.5)
**Age at the first rhGH injection (years)**						
n	1796	209	1585	165	9	406
Mean (SD)	9.4 (3.6)	9.4 (3.8)	9.4 (3.5)	8.0 (3.5)	8.7 (4.7)	8.9 (3.6)
**Patients at first rhGH injection in age classes**						
n	1796	209	1585	165	9	406
<5 years	230 (12.8)	31 (14.8)	199 (12.6)	39 (23.6)	2 (22.2)	73 (18.0)
From 5 to 10 years	732 (40.8%)	80 (38.3%)	651 (41.1%)	77 (46.7%)	5 (55.6%)	163 (40.1%)
>10 years	834 (46.4%)	98 (46.9%)	735 (46.4%)	49 (29.7%)	2 (22.2%)	170 (41.9%)
**Bone age at first rhGH injection (years)**						
n	261	36	225	22	1	65
Mean (SD)	7.6 (3.4)	8.1 (3.7)	7.5 (3.4)	7.3 (3.3)	4.2 (-)	6.9 (3.5)
**Ratio between chronological age and bone age**						
n	261	36	225	22	1	65
Mean (SD)	0.8 (0.2)	0.8 (0.2)	0.8 (0.1)	0.8 (0.1)	0.6	0.8 (0.2)
**Height SDS at baseline**						
n	1745	199	1544	161	9	391
Mean (SD)	-2.5 (0.9)	-2.4 (1.1)	-2.5 (0.9)	-2.7 (0.9)	-3.4 (1.6)	-2.8 (1.0)
**Height velocity at baseline (cm/year)**						
n	515	60	454	32	2	111
Mean (SD)	4.7 (1.8)	4.3 (1.8)	4.8 (1.8)	4.4 (2.2)	5.1 (1.2)	4.8 (2.8)

GHD, growth hormone deficiency; TS, Turner syndrome; CRI, chronic renal insufficiency.

**Table 2:** Patient disposition by subgroups and aetiology – Enrolled population.

	n (%)		
Aetiology	Treatment-naïve patients(N=2381)	Non-treatment-naïve patients(N=407)	All patients(N=2792)^a^
Organic GHD^b^	214 (9.0)^b^	41 (10.1)^d^	255 (9.1)
Pre-pubertal organic GHD	166 (8.8)^c^	–	
Idiopathic GHD^b^	1585 (66.6)^b^	240 (59.0)^d^	1825 (65.4)
Pre-pubertal idiopathic GHD	1240 (65.4)^c^	–	
Turner syndrome^c^	165 (6.9)^b^	34 (8.4)^d^	199 (7.1)
Pre-pubertal Turner syndrome	153 (8.1)^c^	–	
Chronic renal insufficiency^c^	9 (0.4)^b^	1 (0.3)^d^	10 (0.4)
Pre-pubertal chronic renal insufficiency	7 (0.4)^c^	–	
Other non-GHD^c^	406 (17.1)^b^	91 (22.4)^d^	497 (17.8)
Pre-pubertal other non-GHD	329 (17.4)^c^	–	

a Information on treatment-naive status missing for four patients.

b Percentage of total treatment-naïve population.

c Percentage of total treatment-naïve pre-pubertal population.

d Percentage of total non-treatment-naïve population.

### Dosing and Duration of Exposure

The total patient-years of exposure to rhGH in the Enrolled population was 9773. In the Enrolled population, the mean (±SD) dose of rhGH at the study baseline visit was 0.033 (±0.008) mg/kg/day and the median duration of treatment was 38.2 months (3.2 years; range: <1 month–139 months). The starting dose of rhGH varied by indication and followed the label recommendations for each indication (**Supplementary Material Table S1**).

### Effectiveness Results

**Figure 2:**
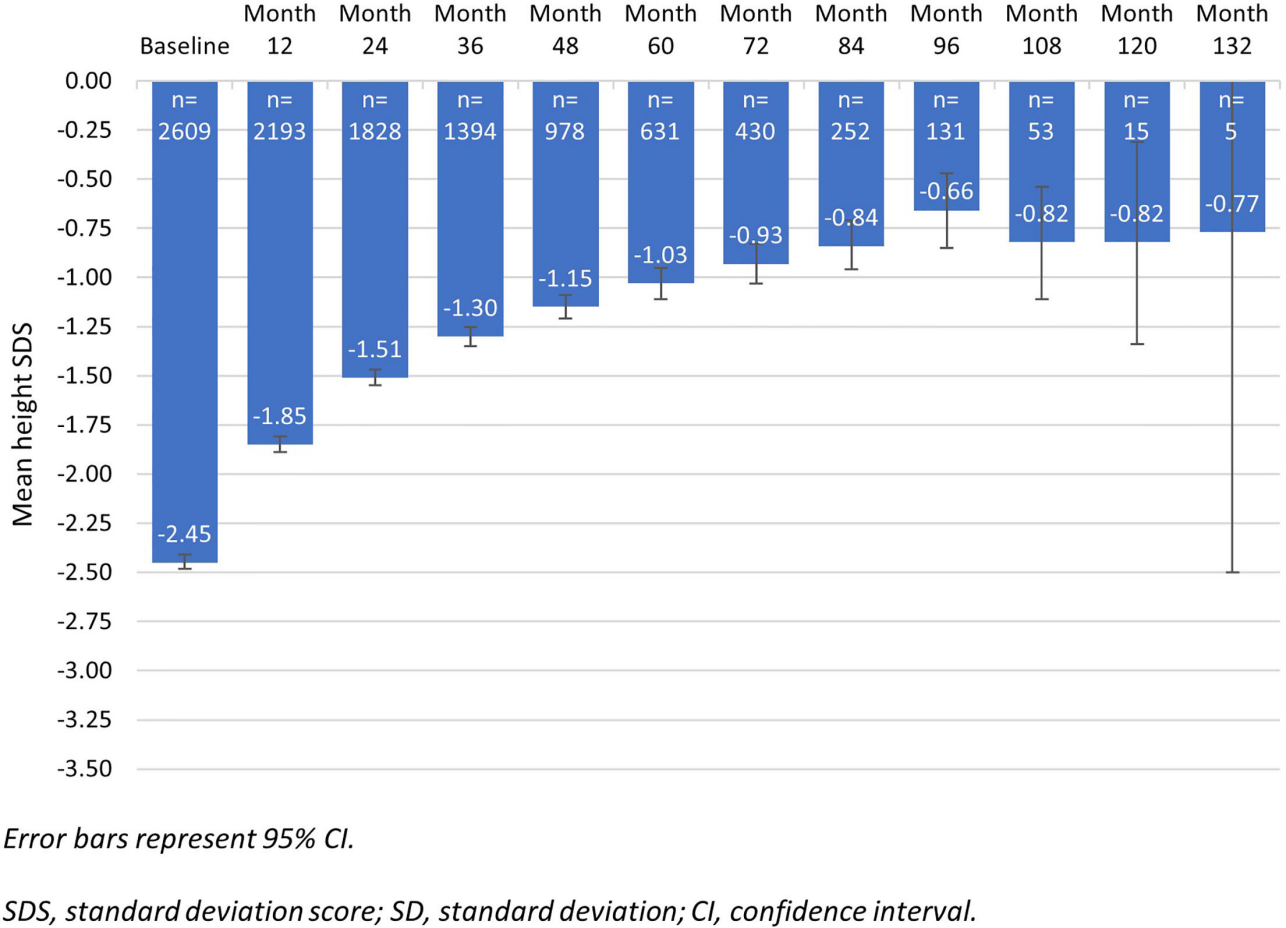
Evolution of height SDS from baseline to month 132 – Registry population (n=2714).

**Figure 3:**
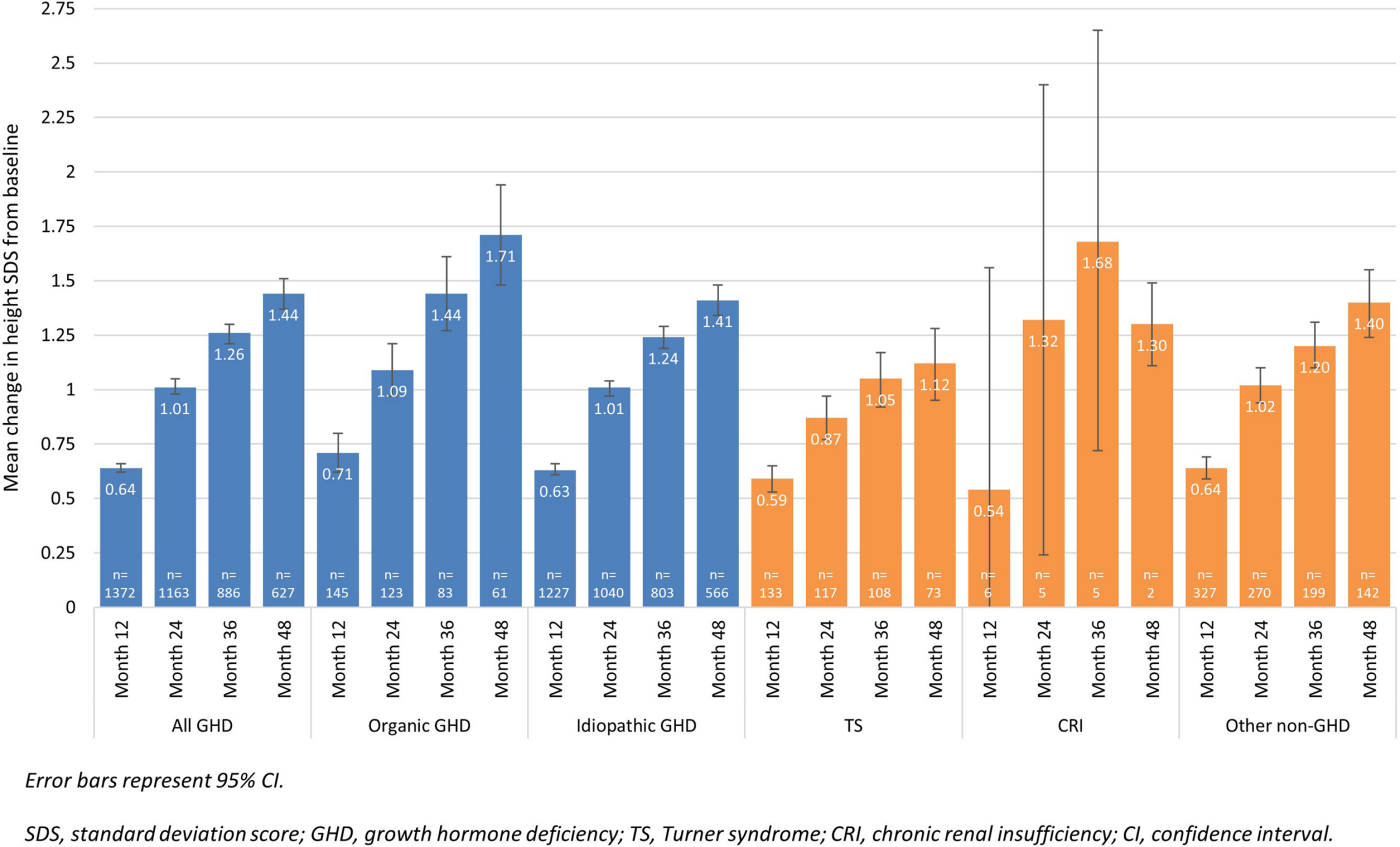
Change in height SDS from baseline in treatment-naïve patients over 48 months, according to disease aetiology – Registry population (n=2314).

After treatment with rhGH, height SDS increased from baseline at each assessment (**Figure 2**; Registry population). At Month 12 the mean (±SD) height SDS had increased from -2.45 (±1.01) at baseline to -1.85 (±0.94) (**Figure 2**). In the treatment-naïve group, there was an increase from baseline in height SDS at all timepoints, irrespective of the aetiology (**Figure 3**). At Month 12, mean (95% CI) change in height SDS from baseline in treatment-naïve patients was 0.60 (0.58;0.62, n=2125): mean change in height SDS at 12 months was 0.63 (0.61;0.66, n=1227) in patients with idiopathic GHD, 0.71 (0.62;0.80, n=145) in those with organic GHD, 0.59 (0.53;0.65, n=133) in TS patients, 0.54 (-0.49, 1.56, n=6) in CRI patients, and 0.64 (0.59;0.69, n=327) in other non-GHD patients. Among treatment-naïve patients with organic GHD, the mean change from baseline in height SDS, from Month 12 to Month 48 increased from 0.71 (95% CI [0.62;0.80], n=145) to 1.71 (95% CI [1.48;1.94], n=61). Among treatment-naïve patients without GHD, the subgroup of patients with TS showed the lowest mean change from baseline in height SDS: from 0.59 (95% CI 0.53;0.65, n=133) at Month 12 to 1.12 (95% CI: 0.95;1.28, n=73) at Month 48. Overall, there was greater improvement in height SDS in patients with GHD than in patients without GHD; the greatest improvement was observed in patients with organic GHD. The greatest mean (±SD) change in height SDS at 12 months compared to baseline was seen in treatment-naïve pre-pubertal patients with organic GHD (0.78 [±0.57], n=113; **Supplementary Material Figure S1**). Results of the univariate analysis were significant at Months 12, 24, 36, and 48 for the parameters of aetiology, pubertal status, biological mother’s height, biological father’s height, target height, height SDS at baseline, weight SDS at baseline, and age at first rhGH intake. In addition, results were significant at Months 12 and 36 for birth length, at Months 12, 24 and 36 for starting dose, and at Month 12 for dose adaptation during year of treatment. Multivariate analysis showed that at Month 12, improvement in height SDS was likely to be better in patients with a high starting dose of rhGH, a high target height, a low height SDS at baseline irrespective of aetiology, or a high birth length. Multivariate analysis results are presented in **Supplementary Material Table S2**. Simulations based on the statistical model showed that for baseline height SDS values lower than -1.5, patients with idiopathic or organic GHD had better improvement in height SDS than patients without GHD. When baseline height SDS was above -1.5, improvement in height SDS was better in patients without GHD, particularly in those with TS.

Mean (±SD) height velocity increased from 5.2 (±2.4) cm/year at baseline (n=806) to 8.5 (±2.2) cm/year after 12 months of rhGH treatment (Registry population; n=2131). The greatest mean height velocity was observed in patients with organic GHD; at Month 12, height velocity had increased from 4.4 cm/year (n=55) at baseline to 9.3 cm/year (n=147). Multivariate analysis showed that at Month 12, height velocity was likely to be higher in patients with a low age at the first rhGH intake, a high birth length, high target height, and a high starting dose regardless of the aetiology.

### Patients Who Reached Adult/Near-Adult Height

For the 235 patients who completed the study and reached adult height or near-adult height, mean height at last visit was 154.0 cm (±8.0) for girls and 166.7 cm (±8.0) for boys, corresponding to an overall mean height SDS of -1.4 ±1.2) for girls and -1.1 (±0.9) for boys, with a mean improvement from baseline of 1.0 (±1.1) for girls and 1.0 (± 0.7) for boys. The mean (±SD) adult height or near-adult height was 5.2 cm ( ± 7.6, n=68) lower than the PAH for boys and 2.8 cm (±2.7, n=76) lower than the PAH for girls. Covariates entered into the linear regression analysis are presented in **Supplementary Material Table S3**. In the multivariate analysis to identify predictive factors for adult or near-adult height SDS in treatment-naïve patients, only height SDS at baseline was statistically significant (p<0.05), with a higher adult height for patients with a higher height SDS at baseline (**Supplementary Material Table S3**).

In the 87 patients in the Enrolled population who ended the study with height satisfaction as the reason for withdrawal, although some were not at adult height, the mean (±SD) height was 129.3 cm (±16.8) at baseline and 160.4 cm (±11.7) at the end of treatment (165.6 cm [±12.7] for boys and 154.0 cm [±6.1] for girls). The mean (±SD) height of patients who ended the study due to height satisfaction and at adult height (n=60) was 4.9 cm (±9.5) lower than the PAH of 165.0 cm (±10.6). Mean ±SD) height SDS improved from -2.3 (±1.0) at baseline to -1.0 (±1.0) at the end of treatment.

### Complementary Additional Analyses Results

In both the treatment-naïve and treatment-naïve pre-pubertal populations of patients with GHD, patients with a GH peak of ≤3 ng/ml had a greater improvement in height SDS at Month 12 than patients with a GH peak of >3 ng/ml (**Supplementary Material Figure S2**). There was a significant difference between GH peak groups for change in height SDS in the treatment-naïve (p=0.036) and treatment-naïve pre-pubertal (p=0.011) populations (ANCOVA analysis).

There were no major differences in baseline characteristics between countries, except that patients appeared to have more severe growth retardation in Germany (lowest height SDS). Prescribed doses of rhGH tended to be highest in France (mean [±SD] 0.038 [±0.008] mg/kg/day in patients with idiopathic GHD).

### Safety

Reports of TEAEs are summarised in **Table 3**. Of the 377 serious TEAEs, the most frequent were pneumonia (9 events) and adenoidal hypertrophy (8 events), and the most frequent related to rhGH was scoliosis (4 events; **Table 3**). TEAEs led to discontinuation in 61 (1.7%) patients. Twelve patients discontinued treatment due to serious TEAEs, five of which were related to rhGH (scoliosis, low-grade astrocytoma, craniopharyngioma, lymphadenopathy and epiphysiolysis). Of the serious TEAEs, 20 patients (0.6%) experienced a malignant or unspecified neoplasm; 17 patients during the treatment period and three patients after stopping treatment. The neoplasms reported as serious TEAEs in the 17 patients during treatment with rhGH were: craniopharyngioma (n=4), recurrent medulloblastoma (n=3), cholesteatoma (n=2), astrocytoma (n=1), low grade astrocytoma (n=1), germ cell cancer (n=1), malignant melanoma (n=1), melanocytic naevus (n=1), ovarian germ cell teratoma (n=1), pituitary tumour (n=1), and teratoma (n=1). Four of these were reported by the Investigator as related to rhGH (low grade astrocytoma, cholesteatoma, craniopharyngioma and germ cell cancer). Of the 20 patients who developed neoplasms reported as serious TEAEs, 5 patients with craniopharyngioma, 4 with medulloblastoma, and 2 with astrocytoma had a history of neoplasm. Three of the four patients with neoplasms reported as serious TEAEs related to rhGH had a medical history of neoplasms. One male patient with no history of neoplasm, developed cholesteatoma approximately 6 years after rhGH therapy initiation, continued on rhGH therapy, and recovered without sequelae after tympanoplasty.

**Table 3:** Overview of frequently reported TEAEs – Safety population (n=3493).

	Number of TEAEs (number of patients; %)^a^	
	Serious	Non-serious
**TEAEs**	377 (206; 5.9%)	NA^b^
Drug-related TEAEs	30 (27; 0.8%)	1070 (610; 17.5%)
**Most common drug-related TEAEs (non-serious events ≥0.3% of patients)**		** **
Insulin-like growth factor increased^c^	–	314 (256; 7.3)
Injection site haematoma	–	99 (86; 2.5)
Injection site pain	–	96 (93; 2.7)
Headache	1 (1; <0.1%)	81 (76; 2.2)
Arthralgia	–	37 (37; 1.1)
Scoliosis	4 (4; 0.1%)	32 (31; 0.9)
Insulin-like growth factor decreased	–	35 (33; 0.9)
Pain in extremity	–	18 (16; 0.5)
Gynaecomastia	–	16 (15; 0.4)
Asthenia	–	15 (15; 0.4)
Glycosylated haemoglobin increased	–	14 (14; 0.4)
Injection site haemorrhage	–	13 (11; 0.3)
Lipodystrophy acquired	–	13 (13; 0.4)
Injection site atrophy	–	9 (9; 0.3)
Blood insulin increased	–	9 (9; 0.3)

a Status of seriousness was not reported for 18 AEs.

b As per the protocol, it was planned to only collect information on SAEs (related and unrelated) and non-serious related AEs. Therefore, as non-serious, unrelated events were not collected, the total number of TEAEs is not applicable.

c As defined by study investigator.

TEAE, treatment-emergent adverse event; NA, not applicable.

A total of seven patients died, three during the study period (streptococcal sepsis; infection; and recurrent medulloblastoma) and four after discontinuation of rhGH (cardio-respiratory arrest; possible central respiratory dysfunction; haematemesis; and dyspnoea). All deaths were considered unrelated to study treatment.

## Discussion

The primary objective of this non-interventional study was to collect long-term safety information on rhGH during treatment of paediatric GH disorders in routine clinical practice. TEAEs were generally consistent with the known safety profile of rhGH. No major or new safety issues were revealed from this study in a large patient population. The risk of neoplasm is included in the product label by regulatory agencies. However, this risk varies according to the underlying cause for GH deficiency (e.g., secondary to intracranial lesion), associated co-morbidities and treatments undertaken (i.e., previous malignant disease or radiation therapy increase this risk) (18). The recurrence of previous neoplasms or secondary neoplasms occurred in <1% of patients in clinical trials (18). The reported rate of neoplasms in the iNCGS of 0.6% is therefore consistent with the experience from clinical trials of this product and registries of other GH products (16, 26). The recent SAGhE cohort study concluded that in patients treated with GH in childhood for isolated GHD, idiopathic short stature or short stature in children born small for gestational age, there was no increased mortality from neoplasms compared with people who had never been treated with GH (27). Craniopharyngioma should be detected if a pre-treatment MRI is conducted, and the classification of craniopharyngioma events in the iNCGS as treatment-related is therefore surprising. However, we include these findings as reported by the investigators as there are no other data to contradict the classification of these events. Furthermore, some events such as cholesteatoma and pituitary tumour (accounting for three of the 17 serious neoplasm events during treatment) were classified as neoplasms in the iNCGS (according to the MedDRA coding), and these ‘neoplasms’ may be benign or malignant.

Results from the study demonstrated that the mean height SDS and mean height velocity were improved in all patient groups with growth failure, whatever their prior treatment status and aetiology. These improvements were evident after 12 months and maintained until Month 48. The subgroup that gained the greatest benefit was treatment-naïve prepubertal patients with organic GHD. Based on multivariate analyses, the improvement in height SDS at Month 12 was better in patients with a higher starting dose, greater target height, lower height SDS at baseline (whatever the aetiology) and greater birth length. These results were also observed at Month 36. Results from both treatment-naïve and treatment-naïve pre-pubertal patients with GHD showed that pre-treatment peak GH may modify the effectiveness of treatment; at Month 12, change in height SDS was greatest in patients with a peak GH of ≤3 ng/ml. Of note, patients with a peak GH of >10 ng/ml were included in the GHD population if the patient was classified as GHD by the physician in charge, despite the threshold for GHD being 10 ng/ml (1).

A total of 235 patients completed the study having attained adult or near-adult height. Mean (±SD) height SDS was -1.1 (±0.9) in boys and -1.4 (±1.2) in girls. Mean (±SD) height at last visit (161.1 cm [±10.2]) corresponded to a mean (±SD) height SDS of -1.2 (±1.0), which is similar to previously-reported effectiveness findings, including results obtained from Canadian patients in a long-term observational study where the mean near-adult height SDS was -1.28 (28). These results suggest that rhGH is effective in improving the adult height of patients with GHD into the normal adult range. Aetiology did not have any significant impact on the adult height, but aetiology did have a significant impact on height SDS change up to 48 months of treatment. This apparent discrepancy may be due to the smaller size of the population completing the study, lacking the statistical power to demonstrate a difference in adult height between aetiologies.

Data from this study suggest that with long-term treatment, rhGH therapy produces a sustained improvement in height velocity and height SDS in patients with all the aetiologies assessed, with the greatest improvement observed in treatment-naïve patients with organic GHD and treatment-naïve pre-pubertal patients with organic GHD. The results in the present study are similar to those seen in controlled clinical trials in patients with GHD (29) as well as in previous long-term observational study settings with GH therapy in patients with GHD or TS (28, 30), suggesting that the efficacy and safety of rhGH is as consistent in the ‘real-world’ setting as it is in the controlled environment of a clinical trial. It should be noted that treatment objectives are not the same for all aetiologies. For example, in patients with GHD and SGA the desired outcome may be to achieve target height, while this may not be the case in for patients with TS.

The study results show improvements with rhGH administration despite a mean of a >1-year delay between initial diagnosis and the start of rhGH treatment. The relationship between latency to treatment onset and the effectiveness of rhGH treatment has not been previously discussed and could be an area for further investigation.

Limitations that are common to observational studies were present in this study. For example, it is difficult in observational studies to assess all patients in the long term, and at least 30% of patients withdrew from the study before completing treatment or before reaching adult height. Thus long-term safety data and/or some outcome parameters may have been affected by these missing data. Furthermore, weight and BMI SDS were collected at baseline but data are not available for these values at end of therapy, which could have provided useful information on the theoretical possibility of a long-term metabolic impact of rhGH treatment. Other protocol-defined endpoints and assessments could be debated – for example, the adult height was compared with predicted adult height but not target height, which may be more important, the final height SDS could have been corrected for mid-parental height, and in the linear regression analysis BMI may be a more relevant covariate than weight SDS. The diagnosis and decision to prescribe rhGH was at the discretion of the treating physician and independent of inclusion in this study, and therefore, diagnostic methodology may not have been applied consistently. For example, the diagnosis of idiopathic GHD was declared by the treating physician but no further diagnostic information was collected due to the non-interventional nature of the study. In addition, biases are possible because of missing data – for example, the proportion of patients switching rhGH formulations and the impact this may have on outcomes is not known. Despite these limitations, this study provides useful information on the long-term effectiveness and safety of rhGH in a ‘real-world’ setting. The aetiologies of short stature included in this study should be representative of those aetiologies in practice (since this study included all eligible patients), but the low number of patients in the CRI group make conclusions difficult for this group. However, since CRI is a rare disease (31, 32), the patient population in this study is representative of real-world practice.

In conclusion, mean height SDS and mean height velocity improved and were sustained in patients with growth failure after treatment with rhGH, regardless of prior treatment status and aetiology. This improvement was greatest in treatment-naïve, treatment-naïve pre-pubertal and treatment-naïve GHD patients, despite a >1-year delay between diagnosis and start of treatment. The data confirm that the benefit-risk profile for rhGH remains favourable, with no new safety concerns, and that rhGH is effective in a real-world setting in children with growth failure who are treated within the recommended indications.

## Data Availability Statement

The datasets presented in this article are not readily available because where patient data can be anonymised, Ipsen will share all individual participant data that underlie the results reported in this article with qualified researchers who provide a valid research question. Study documents, such as the study protocol and clinical study report, are not always available. Proposals should be submitted to DataSharing@Ipsen.com and will be assessed by a scientific review board. Data are available beginning six months and ending five years after publication; after this time, only raw data may be available. Requests to access the datasets should be directed to DataSharing@Ipsen.com.

## Ethics Statement

The studies involving human participants were reviewed and approved by all relevant Ethics Committees and Institutional Review Boards. Written informed consent to participate in this study was provided by the participants’ legal guardian/next of kin.

## Author Contributions

All authors contributed to the article and approved the submitted version.

## Funding

This study was sponsored by Ipsen.

## Conflict of Interest

RC received honoraria for lectures and for the scientific organization of meetings from Sandoz, Ipsen, Novo Nordisk, Lilly, and Pfizer. JB-M received honoraria for lectures from Lilly, Merck Ferring, Sandoz and Ipsen and participated in Ipsen advisory board. DS served in advisory boards for Kyowa Kirin, Ipsen, Merck Serono, Novo Nordisk; received honoraria for lectures and for the scientific organization of meetings from, Hexal/Sandoz Ipsen, Merck Serono, Novo Nordisk and Pfizer; and received research grants from Hexal/Sandoz, Kyowa Kirin and Pfizer. MD received honoraria for lectures and for the scientific organization of meetings from Sandoz, Ipsen, Novo Nordisk and Pfizer. CS and VP are employees of Ipsen.

The authors declare that this study received funding from Ipsen. The funder contributed to study design, data collection and analysis, decision to publish, and funded editorial support for preparation of the manuscript. The authors take full responsibility for the entire content of this submitted manuscript and approved submission.

The remaining author declares that the research was conducted in the absence of any commercial or financial relationships that could be construed as a potential conflict of interest.
