# Supplementary material for: Effectiveness and Overall Safety of NutropinAq® for Growth Hormone Deficiency and Other Paediatric Growth Hormone Disorders: Completion of the International Cooperative Growth Study, NutropinAq® European Registry (iNCGS)
Source: Front Endocrinol (Lausanne). 2021 May 25;12:676083. doi: 10.3389/fendo.2021.676083 (PMC8185283; doi:10.3389/fendo.2021.676083)
Supplement: Supplementary file 1 [file DataSheet_1.docx]

**Supplementary Materials**

**Table S1: Exposure to rhGH – Enrolled population**

| **Enrolled population** | **Idiopathic GHD (n=1825)** | **Organic GHD** **(n=255)** | **TS** **(n=199)** | **CRI (n=10)** | **Other non‑GHD**  **(n=498)** |
| --- | --- | --- | --- | --- | --- |
| **Treatment duration (months)** |  |  |  |  |  |
| n | 1825 | 250 | 199 | 10 | 498 |
| Mean (SD) | 42.2 (26.5) | 40.1 (29.3) | 48.3 (29.0) | 40.8 (35.4) | 40.4 (26.9) |
| Median (min;max) | 39.6 (0;139) | 32.0 (0;124) | 43.5 (0;128) | 35.3 (0;103) | 34.8 (0;123) |
| **Initial dose (mg/kg/day)** |  |  |  |  |  |
| n | 1790 | 244 | 195 | 9 | 492 |
| Mean (SD)  Median (min;max) | 0.032 (0.007)  0.031 (0.010;0.094) | 0.030 (0.008)  0.030 (0.000;0.067) | 0.043 (0.009)  0.043 (0.022;0.077) | 0.042 (0.010)  0.044 (0.028;0.059) | 0.034 (0.009)  0.034 (0.012;0.066) |

GHD, growth hormone deficiency; TS, Turner syndrome; CRI, chronic renal insufficiency; SD, standard deviation.

**Table S2: Multivariate analysis for change in height SDS from baseline to Months 12, 24, 36 and 48 in treatment‑naïve patients – Registry population**

|  | **Month 12** | | **Month 24** | | **Month 36** | | **Month 48** | |
| --- | --- | --- | --- | --- | --- | --- | --- | --- |
|  | Estimate [95% CI] | p-value | Estimate [95% CI] | p-value | Estimate [95% CI] | p-value | Estimate [95% CI] | p-value |
| ***Multivariate analysis*** | | | | | | | | |
| Height SDS at baseline | -0.538 [-0.789;-0.286] | <0.001 | -0.280 [-0.324;-0.235] | <0.001 | -0.241 [-0.296;-0.186] | <0.001 | -0.352 [-0.409;-0.294] | <0.001 |
| Age at first rhGH intake (years) | NS | – | NS | – | -0.079 [-0.096;0.062] | <0.001 | NS | – |
| Birth length (cm) | 0.034 [0.017;0.051] | <0.001 | NS | – | 0.028 [0.016;0.040] | <0.001 | NS | – |
| Starting dose (μg/kg/day) | 8.761 [5.306;12.216] | <0.001 | 14.183 [9.981;18.386] | <0.001 | 16.588 [10.131;23.046] | <0.001 | NS | – |
| Target height (cm) | 0.005 [0.002;0.008] | 0.002 | 0.008 [0.005;0.012] | <0.001 | 0.009 [0.004;0.015] | <0.001 | 0.015 [0.009;0.021] | <0.001 |
| **Pubertal status**  Pubertal (reference)  Pre-pubertal | NS  NS | –  – | 0.00  0.110 [0.031;0.188] | 0.006 | NS  NS | –  – | NS  NS | –  – |
| **Aetiology**  Idiopathic GHD (reference)  Organic GHD  Other non-GHD  Turner syndrome | 0.00  0.133 [-0.092;0.357]  0.259 [0.029;0.488]  0.377 [0.072;0.683] | 0.021 | 0.00  0.343 [0.080;0.605]  0.299 [0.067;0.532]  0.073 [-0.323;0.468] | 0.014 | 0.00  0.190 [-0.250;0.629]  -0.154 [-0.526;0.219]  -1.115 [-1.524;0.705] | <0.001 | 0.00  0.335 [0.140;0.531]  -0.077 [-0.222;0.068]  -0.274 [-0.463;-0.086] | <0.001 |
| **Interaction between birth length and height SDS at baseline** | 0.007 [0.002;0.012] | 0.006 | NS | – | NS | – | NS | – |
| **Interaction between age at first rhGH intake and aetiology**  Idiopathic GHD (reference)  Organic GHD  Other non-GHD  Turner syndrome | NS  NS  NS  NS | –  –  –  – | NS  NS  NS  NS | –  –  –  – | 0.00  0.009 [-0.037;0.054]  -0.000 [-0.041;0.040]  0.083 [0.037;0.128] | 0.005 | NS  NS  NS  NS | –  –  –  – |
| **Interaction between height SDS at baseline and aetiology**  Idiopathic GHD (reference)  Organic GHD  Other non-GHD  Turner syndrome | 0.00  0.029 [-0.053;0.112]  0.112 [0.033;0.191]  0.203 [0.097;0.309] | <0.001 | 0.00  0.094 [-0.001;0.190]  0.146 [0.066;0.266]  0.151 [0.014;0.289] | 0.001 | NS  NS  NS  NS | –  –  –  – | NS  NS  NS  NS | –  –  –  – |

CI, confidence interval; GHD, growth hormone deficiency; NS, not significant; rhGH, recombinant human growth hormone; SDS, standard deviation score.

**Table S3: Univariate and multivariate analysis for final height SDS in treatment‑naïve patients who reached adult/near‑adult height – Complete population**

| **Covariates** | **Univariate analysis** | **Multivariate analysis** | |
| --- | --- | --- | --- |
|  | p-value | Estimate [95% CI] | p-value |
| Aetiology | 0.006 | – | NS |
| Gender | 0.011 |  | NS |
| Pubertal status | NS | – | NS |
| Biological mother height | 0.003 | – | NS |
| Biological father height | <0.001 | – | NS |
| Birth length | 0.035 | – | NS |
| Target height | <0.001 | – | NS |
| Height SDS at baseline | <0.001 | 0.597 [0.457; 0.718] | <0.05 |
| Weight SDS at baseline | <0.001 | – | NS |
| Starting dose (mg/kg/day) | 0.086 | – | NS |
| Mean dose | NS | – | NS |
| Age at first rhGH intake | NS | – | NS |

NS, not significant; rhGH, recombinant human growth hormone; SDS, standard deviation score

**Fig. S1: Change from baseline in height SDS at 12 months**

**
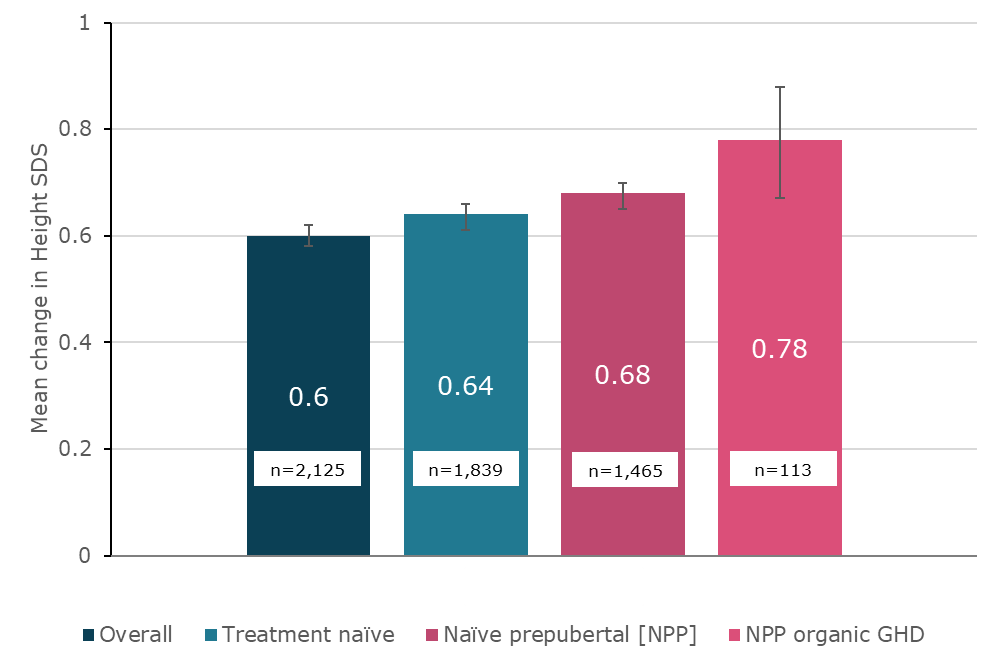
**

*Error bars represent 95% CI*

SDS, standard deviation score; NPP, treatment‑naïve pre‑pubertal; GHD, growth hormone deficiency.

**Fig. S2: Effectiveness in GHD patients at 12 months by GH peak in mean change in height SDS from baseline**

**
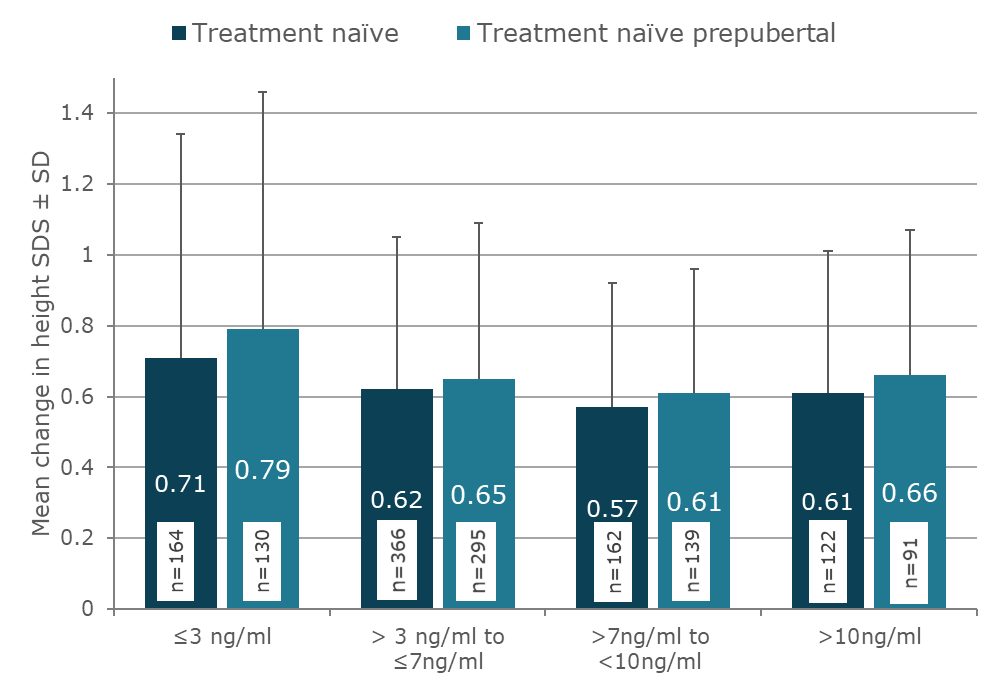
**

*Error bars represent ±SD*
